# Reducing the Flocculation of Milk Tea Using Different Stabilizers to Regulate Tea Proteins

**DOI:** 10.3390/foods12071484

**Published:** 2023-04-01

**Authors:** Yuqi Song, Xiaosen Wang, Haixi Luo, Mingyan Wang, Jian Chen

**Affiliations:** 1 Key Laboratory of Food Nutrition and Functional Food of Hainan Province, Engineering Research Center of Utilization of Tropical Polysaccharide Resources, Ministry of Education, College of Food Science and Technology, Hainan University, Haikou 570228, China; 2 Key Laboratory of Medicinal and Edible Plant Resources of Hainan Province, Hainan Vocational University of Science and Technology, Haikou 571126, China

**Keywords:** milk tea, stability, flocculation control, characterization of features, precipitation envelope

## Abstract

The regulation of flocs derived from polyphenol–protein formation in milk tea has not been fully explored. In this study, the flocculation of milk tea was regulated by adding 10 kinds of stabilizers with different characteristics. The stability coefficient and centrifugal precipitation rate were used as indexes. The optimal concentration ratio of the complex stabilizer was identified using the response surface methodology (RSM), being 0.04% for Arabic gum, 0.02% for β-cyclodextrin and 0.03% for Agar. Fourier transform infrared spectroscopy (FT-IR), X-ray diffraction (XRD) and scanning electron microscopy (SEM) were used to analyze the characteristics of different stabilizers in milk tea, and our findings were as follows: (1) The relative strength of the peaks in different stable systems was different. The absorption peaks were mainly near the wave numbers 3376 cm^−1^, 2928 cm^−1^, 1655 cm^−1^, 1542 cm^−1^, 1408 cm^−1^, 1047 cm^−1^ and 925 cm^−1^. (2) The milk tea system was an amorphous structure. The diffraction peak of the composite system was observed to be about 20°. The crystallinity of the milk tea in the compound group was 33.16%, which was higher than that of the blank group (9.67%). (3) The compound stabilizer reduced flocculation, and the stabilizing agents improved the surface order of milk tea. These results indicate that the combination of polysaccharide stabilizers (Arabic gum and agar) and oligosaccharide stabilizers (β-CD) in certain proportions can regulate the flocculation of milk tea and improve its stability. The potential research avenues involving polyphenol–protein complex instability systems and their applications in food development are expanded by this work.

## 1. Introduction

Tea is a good natural drink. The main components of tea include polyphenols and caffeine. In addition, it contains many trace ingredients. Tea lowers the risk of cardiovascular diseases and other health conditions [1]. When fresh milk is processed into milk powder, the moisture content in milk is greatly reduced. Most nutrients are concentrated, thereby preserving protein, fat and other vitamins and micronutrients. Commercially available milk tea is divided into liquid and solid categories. Liquid milk tea is composed of milk and tea juice, while solid milk tea refers to tea powder and milk powder used as raw materials through dry mixing or drying [2].

However, milk tea is a complex and dispersed system composed of many ingredients [3]. During its preparation, due to the interaction of tea polyphenols with proteins, compounds can easily flocculate and precipitate [4]. Even solid milk tea flocculates and forms polyphenol–protein complexes after long-term storage [5,6]. The interaction of small polyphenols with protein mainly occurs on proline and arginine residues of the peptide chain. A small polyphenol can bind to an amino acid residue. Each of the macromolecular polyphenols, such as epigallocatechin gallate (EGCG), binds to an average of 7.5 proline residues at binding equilibrium. These results indicate an obvious steric hindrance when complex polyphenols interact with proteins. When the polyphenol and protein complex reach a certain degree of binding, the polypeptide dimer precipitates as an insoluble aggregate [3,7]. Song and Yoo used EGCG/polyproline as an experimental model. It was found that with the increase in the polyphenol concentration, the coated degree of the polyphenol increased. When the coating is large enough to form a polyphenol–polyphenol bridge, the protein dimerizes and precipitates [8]. Jauregi et al., suggested that whey protein can be used to improve food/beverage formulations containing bioactive ingredients. Whey proteins have very interesting properties including high solubility in water, strong interactions with polyphenols and aggregation properties, and they are classified as GRAS [9].

Moreover, the regulation of the flocculation of milk tea has not been systematically studied. Thongkaew et al., compared the interactions of four kinds of polyphenols with whey protein isolate and a whey protein–pectin complex. It was found that the type and concentration of polyphenols did not affect the particle size and potential after interaction with the whey protein isolate or protein–pectin complex. However, the polyphenols precipitated when added to the whey protein isolate but not when added to the protein–pectin complex [10]. Jongberg et al., characterized protein–polyphenol interactions during the forced aging of beer. The proteins and proteinaceous material were extracted through the acetone precipitation of beer that had undergone forced aging. Treatment of the crude beer extract with sulphite (2M) dissociated the protein–polyphenol bonds of lipid transfer protein 1 (LTP1) and Protein Z, which were generated during moderate forced aging [4].

Therefore, the experimental team added stabilizers with different characteristics to milk tea in order to regulate the flocculation of the polyphenol–protein complex. Polysaccharides can inhibit droplet aggregation and flocculation through electrostatic interactions and steric hindrance and are widely used in the food and cosmetic industries in forms such as xanthan gum, carrageenan and Arabic gum [11,12,13]. β-cyclodextrin is a naturally occurring cyclic oligosaccharide with limited water solubility (1.8%). It is considered an ideal candidate for complexation because it bears a perfect cavity size, efficient loading and drug complexation, high availability and a low cost [14]. Agar is a heterogeneous complex mixture of related polysaccharides sharing the same backbone chain structure. As a thickening agent, agar carries several hydrophilic groups in its molecular structure, such as hydroxyl and carboxylic acid groups [15,16,17]. Sodium alginate, guar gum and sodium carboxymethyl cellulose are also used for increasing food stability, and they all feature high viscosity. Guar gum also increases the water-soluble fiber content of the foods it is added to [18,19,20]. Both glycerol monostearate and sucrose esters have surface-active effects and can reduce surface tension. They also have good emulsification-, dispersion-, foaming- and viscosity-enhancing properties [21,22,23,24].

To further study the regulation of polyphenol–protein flocculation in milk tea, in this study, 10 different stabilizers were added to milk tea to adjust its flocculation, while a blank milk tea group was used as the control to evaluate the stabilizing effects of different stabilizers on milk tea. The response surface methodology (RSM) was applied to the three selected stabilizers to obtain the ratios of the complex stabilizers. In addition, to the best of our knowledge, there have been no previous reports regarding the effects of stabilizers in regulating the characterization of milk tea. Therefore, in this study, Fourier infrared spectroscopy (FT-IR), X-ray diffraction (XRD) and scanning electron microscopy (SEM) were used to analyze the characteristics of single stabilizers and complex stabilizers in the milk tea system.

## 2. Materials and Methods

### 2.1. Reagents and Materials

Skim milk powder was purchased from Inner Mongolia Yili Industrial Group Co., Ltd. (Hefei, China). Ceylon high-quality black tea powder was purchased from Hangzhou Mingbao Biotechnology Co., Ltd. (Suzhou, China). White granulated sugar was purchased from a local supermarket (Haikou, China). The following food stabilizers were purchased from Henan Wanbang Chemical Technology Co., Ltd. (Zhengzhou, China): xanthan gum, carrageenan, sodium carboxymethyl cellulose (CMC), acacia gum, guar gum, sodium alginate (SA), β-cyclodextrin (β-CD), agar, glycerol monostearate (GMS) and sucrose esters (SE). The instruments were as follows: a table-top high-speed refrigerated centrifuge (5805, Eppendorf, Hamburg, Germany); analysis balance (AUW220, Shimadzu Enterprise Management Co., Ltd., Shanghai, China); vacuum Freeze dryer (Alpha 1-4 LD Plus, Marin Christ, Osterode, Germany); microplate Reader (Synergy LX, BMG Labtech, Offenburg, Germany); and field emission scanning electron microscope (Verios G4 UC, Thermo Fisher Technology Co., Ltd., Shanghai, China).

### 2.2. Study of Different Stabilizers

#### 2.2.1. Sample Preparation

Milk tea samples were simulated by using 1 g black tea powder, 6 g skim milk powder and 3 g sugar as raw materials [25]. At different concentrations (0.01%, 0.02%, 0.03%, 0.04% and 0.05%), 10 kinds of stabilizing agents, such as xanthan gum and carrageenan, were added to milk tea powder for dry mixing (the stabilizer ratios were discovered through multiple sets of pre-experiments), using a ratio of milk tea powder: hot water (85–90 °C) of 1:10 for brewing and constant stirring during the brewing process until the fluids were fully mixed [26]. After quiet placement, the single-factor test was carried out with the stability coefficient and centrifugal precipitation rate as two indexes.

#### 2.2.2. Emulsion Stability Coefficient (R) Value

After emulsifying the sample with stabilizer, it was diluted 100 times with deionized water. The absorbance of the sample was measured at a wavelength of 750 nm using the enzyme marker and recorded as *A*_1_. The same sample was diluted 100 times with deionized water after 4500 rpm and 15 min later, and the absorbance of the sample was recorded as *A*_2_ at the same wavelength. Milk tea without stabilizer was used as a blank control group, and three parallel experiments were conducted [27,28]. The stability coefficient formula is as follows:

$$R=\frac{A_{2}}{A_{1}}$$

#### 2.2.3. Centrifugal Precipitation Rate (S%)

The samples of milk tea with different concentrations of stabilizer were weighed and denoted as *M*_1_. The samples were centrifuged at 4 °C and 8000 rpm for 15 min. After the supernatant was poured out, the mass of the precipitate was measured as *M*_2_. The centrifugal precipitation rates of the 10 groups of different stabilizer schemes were determined. The milk tea without stabilizer was used as a blank control group, and three parallel experiments were carried out [29]. The formula for the centrifugal precipitation rate is as follows:

$$S(\%)=\frac{M_{2}}{M_{1}}\times 100\%$$

#### 2.2.4. Response Surface Analysis (RSM)

The response surface methodology is an established and reasonable technology for the identification of the optimal process condition using a multivariate quadratic equation fitting the experimental factors and results [30]. The control level is usually determined in the previous single-factor conditional experiment. Then, one selects a feasible range to construct the RSM and validate its accuracy.

The selection of additives and the proportion of mixture are important factors affecting the stability of milk tea. The compound stabilizer can play a synergistic role. On the basis of the results of the single-factor test, the factor level was determined, and the Box–Behnken test was designed to incorporate 3 factor levels (Supporting Information, Table S1). Then, response surface analysis was used to obtain the optimal compound stabilizer amount for addition. Experiments were randomly run, and duplicate analyses were performed at each design point. The regression analysis results, statistical significance and response surface were analyzed.

### 2.3. Characterization of Milk Tea under the Influence of Different Stabilizers

#### 2.3.1. Fourier Infrared Spectroscopy (FT-IR) Determination

The samples were pre-frozen at −40 °C for 24 h and then vacuum-freeze-dried. After drying, the sample was ground into powder and placed on a drying dish for later use. During the measurement, an appropriate amount of sample powder was placed on the disc pressing tablet for measurement. The scanning spectrum range was 4000–400 cm^−1^, the resolution was 4 cm^−1^, and the scanning time was 64. The infrared spectrum of the sample was recorded [31].

#### 2.3.2. X-ray Diffraction (XRD) Determination

The sample was vacuum-freeze-dried according to the method described in Section 2.3.1 and then ground into a fine powder to be tested. The appropriate amount of sample powder was placed on the slide, pressed and then tested using an X-ray diffractometer. The scan range was 5–60° (2ɵ), with a scanning speed of 5°/min [32]. The atlas of the sample was recorded, and the crystallinity of the sample was calculated using Origin2019 based on the measurement results, as follows:

$$\mathrm{Degree}\;\mathrm{of}\;\mathrm{crystallinity}(\%)=\frac{\mathrm{The}\;\mathrm{area}\;\mathrm{of}\;\mathrm{the}\;\mathrm{crystallization}\;\mathrm{peak}}{\mathrm{The}\;\mathrm{total}\;\mathrm{area}\;\mathrm{of}\;\mathrm{all}\;\mathrm{peaks}\;\mathrm{in}\;\mathrm{the}\;\mathrm{crystalline}\;\mathrm{and}\;\mathrm{amorphous}\;\mathrm{regions}}\times 100\%$$

#### 2.3.3. Scanning Electron Microscopy (SEM) Detection

The sample was prepared for use, baked dry, sputtered and gilded. Before observation, the sample was fixed on the sample copper platform, and the vacuum degree was 1.5 × 10^−3^ Pa. The samples were subjected to an electron microscope for observation and photographed at 5 kV accelerated voltage. After obtaining SEM images of the micromorphology of the milk tea supplemented with different stabilizers (5000×, 10,000× and 20,000×), representative areas were selected for shooting [33].

## 3. Results and Discussion

### 3.1. Effects of Different Stabilizers on the Stability of Milk Tea

**Figure 1:**
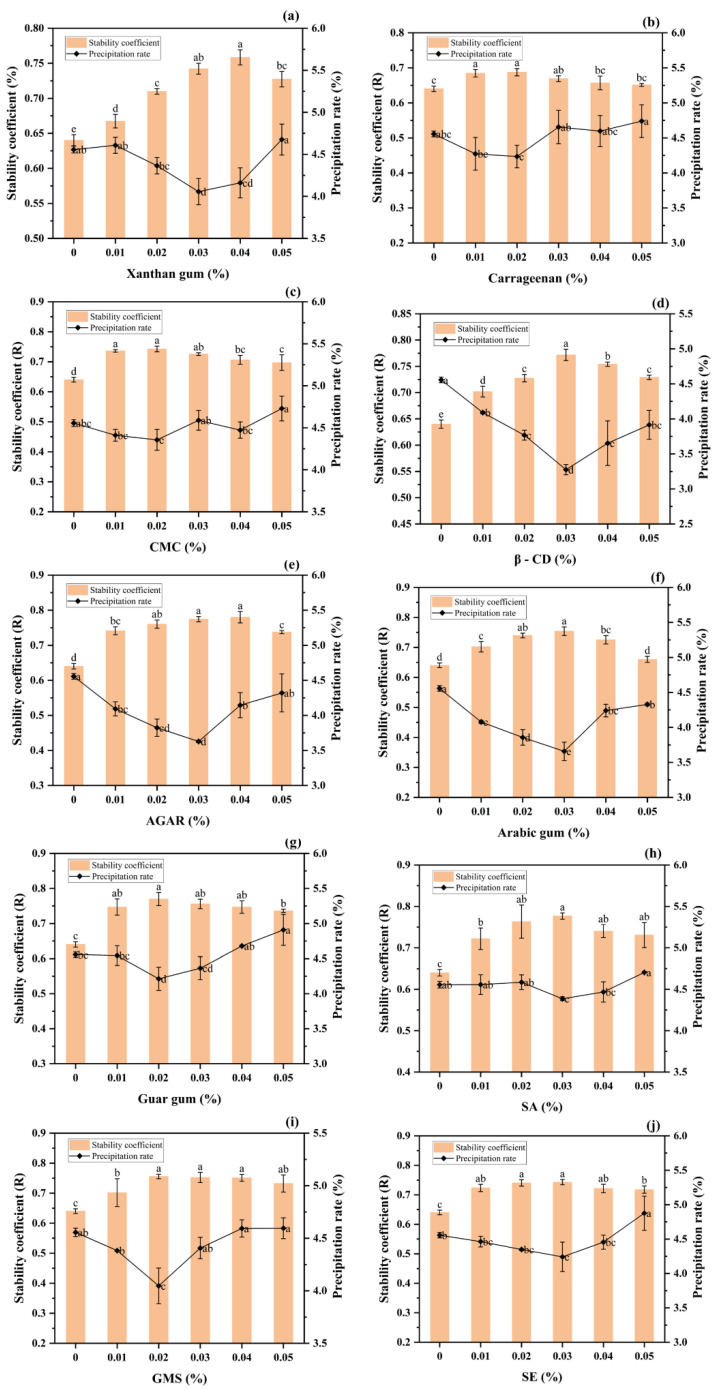
Single-factor test of different stabilizers (data represent mean ± SD, and the different letters on the columns indicate a significant difference at *p* < 0.05). (**a**) Xanthan gum; (**b**) Carrageenan; (**c**) Sodium carboxymethyl cellulose (CMC); (**d**) β-cyclodextrin (β-CD); (**e**) AGAR; (**f**) Acacia gum; (**g**) Guar gum; (**h**) Sodium alginate (SA); (**i**) Glycerol monostearate (GMS); (**j**) Sucrose esters (SE).

Ten different stabilizers were added into the milk tea, and the single-factor test was conducted with the stability coefficient (R) and centrifugal precipitation rate (S) as the determination indexes, as shown in Figure 1 below. If the milk tea group supplemented with stabilizers exhibited a high stability coefficient and low centrifugal precipitation rate and was considered to have better properties than the blank milk tea control group, this indicated that the type and content of added stabilizer(s) had positive effects on flocculation.

According to the figure, the 10 stabilizers enhanced the stability of the milk tea. Xanthan gum (Figure 1a), carrageenan (Figure 1b) and CMC (Figure 1c) are the most common thickeners. They are highly viscous and exhibit thickening, suspending, emulsifying and stabilizing properties in beverages. The stability coefficient first increased and then decreased. The precipitation rate of xanthan gum continued to rise after the addition of 0.03%. However, the precipitation rates of (Figure 1b,c) fluctuated. This may be because the electrostatic action of the xanthan gum did not inhibit droplet aggregation, and like carrageenan and CMC, its high viscosity led to more precipitates. β-CD (Figure 1d), agar (Figure 1e) and Arabic gum (Figure 1f) are good stabilizers. They have high stability coefficients and low precipitation rates. The precipitation rate of β-CD is the lowest, at 3.275%, and better than that of the control group and other stabilizers. It is likely that because β-CD is hollow and cylindrical in structure, being hydrophilic on the outside and hydrophobic on the inside, it can prevent polyphenol–protein binding and flocculation due to its special cavity size. Agar also plays a role in stabilizing milk tea.

Because Agar is a hydrophilic colloid, it prevents the precipitation of whey via the formation of hydrogen bonds [34]. After hydration with water molecules, some hydrophilic groups are highly dispersed in water in a molecular state, forming a single-phase uniform dispersion with high viscosity. Agar’s stability coefficient is not significantly different compared with those of the control group and β-CD, despite its higher precipitation rate. Arabic gum is a polysaccharide secreted by plants. It is a stabilizer that shows good surface activity in milk tea. This may be because the arabinogalactan protein (AGP) in Arabic gum specifically causes emulsification in milk tea [35]. Its hydrophilic sugar residues and hydrophobic amino acids contribute to the interfacial activity of milk tea, thus achieving its stabilization. Here, the centrifugal precipitation rates of guar gum (Figure 1g) and sodium alginate (Figure 1h) were relatively high. This may be because the viscosity of these two stabilizers is too great in milk tea and, therefore, the particles are not dissolved in time, easily separate and, ultimately, precipitate. Consequently, these stabilizers had no significant effects on the stability of the milk tea system. Glycerol monostearate (Figure 1i) and sucrose esters (Figure 1j) are common emulsifiers, and when used at concentrations of 0.02–0.03%, they improve the stability of milk tea. Their poor performance relative to other stabilizers may be due to the low lipid content of the experimental milk powder, resulting in poor emulsion stability.

The single-factor test showed that when 0.03% Arabic gum, 0.03% β-CD and 0.03% agar were added to the milk tea, the stability coefficients and centrifugal precipitation rates were better than those resulting from the other stabilizers. Therefore, response surface analysis was carried out for these three stabilizers.

### 3.2. Comparison of the Optimal Concentrations of Single-Factor Stabilizer

**Figure 2:**
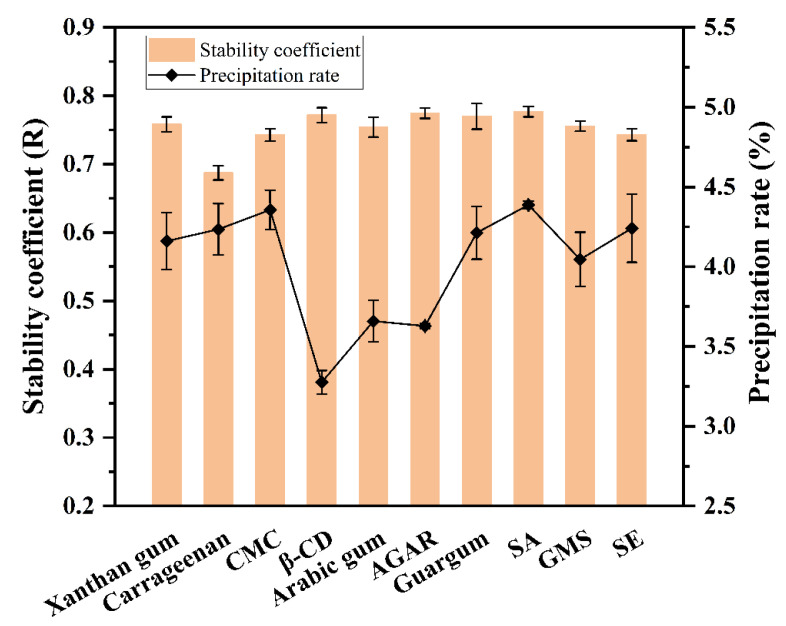
Optimal single-factor comparison of stabilizers.

The optimal concentrations of various stabilizers were compared, as shown in Figure 2. It was found that β-CD, agar and Arabic gum showed the best stability coefficients and the lowest precipitation rates in milk tea. Their immediate solubility is better than that of the other stabilizers, probably because the structural differences in polysaccharide regulate the interaction between protein and polyphenols. The stability coefficients of the three stabilizers decreased in the following order: agar > β-CD > Arabic gum. The precipitation rate increased as follows: β-CD < agar < Arabic gum. Therefore, RSM analysis was carried out to determine the dosage ratio of the compound stabilizer.

### 3.3. RSM Analysis

#### 3.3.1. Results of RSM and Simulated Variance Analysis

The response surface test was conducted according to Section 2.2.4, and the stability coefficient (R) was used as the judgment index to determine the amount of complex stabilizer for addition. Based on the principle of a Box–Behnken central composite design, a regression equation was formulated. Multiple linear regression and binomial fitting analysis were carried out based on the results of the RSM, and the regression equation was obtained as follows: Y = 0.76 + 0.18X_1_ − 0.011X_2_ − 0.006056X_3_ − 0.009591X_1_X_2_ + 0.013X_1_X_3_ − 0.010X_2_X_3_ − 0.026X_12_ − 0.009793X_22_ − 0.017X_32_.

The test results and simulation analysis of variance are shown in Tables S2 and S3 below (Supporting Information, Tables S2 and S3). As shown in Table S3, F = 38.27 in the established regression model (*p* < 0.0001) indicates highly significant differences. However, the P of the missing fitting term was 0.6226, and therefore, the model difference was not significant. This shows that the equation is reliable. The model correlation coefficient R = 0.9801 indicates that the test model fits the actual test perfectly. Approximately 98.01% of the results of the actual test can be explained by the fitting model. After correction, the determination coefficient R^2^_Adj_ was 0.9545, which was close to R, suggesting the sufficient accuracy and universality of the model.

#### 3.3.2. Box–Behnken Response Surface Analyses

**Figure 3:**
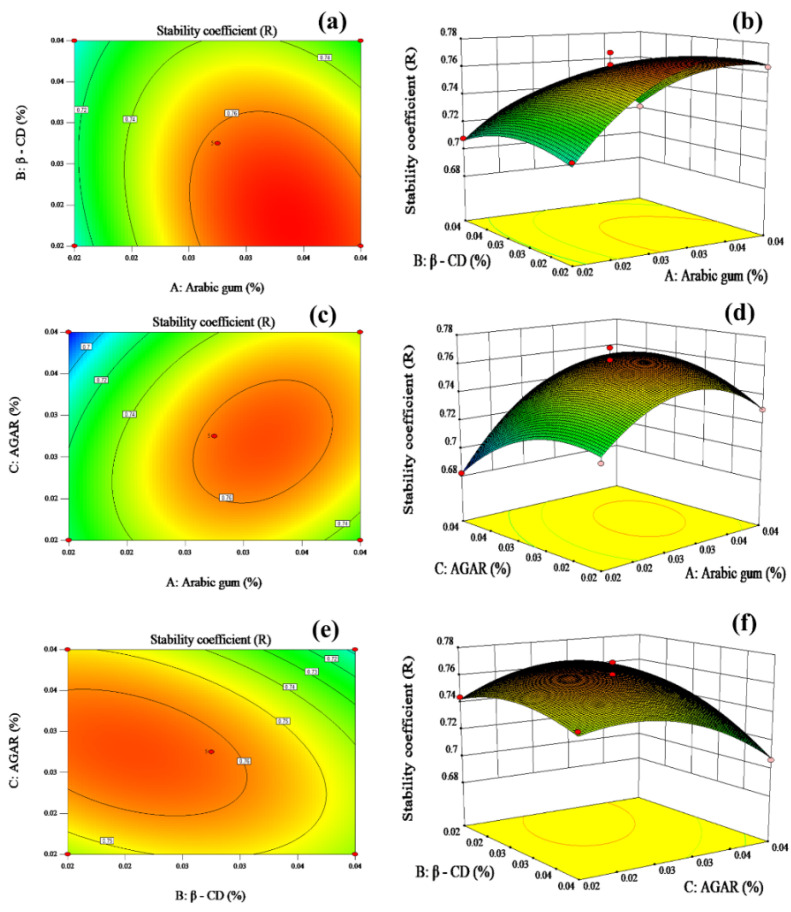
Influence of interaction between various factors on the stability of milk tea. 2-D contour plots and 3-D response surface of β-CD and Acacia gum (**a**,**b**), AGAR and Acacia gum (**c**,**d**), AGAR and β-CD (**e**,**f**).

Based on the results of the variance analysis of regression model, the response surface and contour maps were drawn. The effects of acacia gum, β-CD and agar on the stability of milk tea were determined. When one of these three factors was fixed, the impact of the interaction between the other two factors on the response value was represented using contour and response surface diagrams, as shown in Figure 3.

The response surface and contour maps directly reflect the effect of the interaction on the response value. The steeper the surface and the denser the contour are, the more significant the effect is. The closer the contour line is to the ellipse, the stronger the interaction between the two factors is [30,36]. According to Figure 3a,c,e, the interaction between β-CD and agar was greater than the interaction between β-CD and Arabic gum, which was stronger than the interaction between Arabic gum and agar. According to Figure 3b, the addition of small amounts of β-CD increased the stability of milk tea with the increase in the amount of Arabic gum. The addition of high levels of β-CD first increased the stability of milk tea, followed by eventual decline with the addition of Arabic gum. According to Figure 3d, the addition of small amounts of acacia gum decreased the stability of milk tea with the increase in the amount of agar. The addition of large amounts of acacia gum first increased the stability of milk tea, followed by a slow decrease with the addition of agar. According to Figure 3f, the stability of milk tea first increased and then decreased with the addition of β-CD, following the addition of small amounts of agar. The stability of milk tea decreased with the increase in β-CD when the amount of agar was increased.

#### 3.3.3. Verification Test

The regression equation was implemented using the stability coefficient R as the index. The optimal stabilizers were acacia gum (0.04%), β-CD (0.02%) and agar (0.03%). The best formula was verified, and three parallel tests were set up. The mean value of R was 0.774648, which was similar to the theoretical value of 0.772292. Therefore, this was considered as the best scheme.

### 3.4. Analysis of Characterization

#### 3.4.1. Fourier Infrared Spectroscopy (FT-IR) Analysis

**Figure 4:**
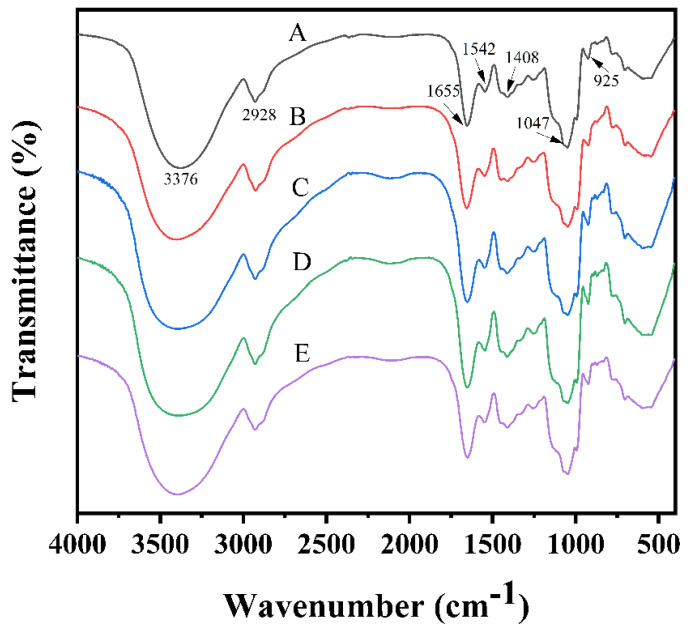
Infrared spectra of milk tea subjected to different stabilizers.

Vibrational spectra are directly related to the structural features of molecules. FTIR can determine the frequency of bond vibrations. The different effects of the milk tea samples with different stabilizers and blank milk tea samples on the milk tea system were observed by infrared spectroscopy. Figure 4 shows the infrared spectra of the milk tea after treatment with different stabilizers. In the figure, A denotes milk tea without stabilizer (blank group); B, C and D represent milk tea supplemented with 0.03% acacia gum, β-CD and agar, respectively; and E was treated with the optimal complex stabilizer obtained from the response surface.

The common absorption peaks in the infrared absorption spectra of A−E are mainly near the wave numbers 3376 cm^−1^, 2928 cm^−1^, 1655 cm^−1^, 1542 cm^−1^, 1408 cm^−1^, 1047 cm^−1^ and 925 cm^−1^. The wide peak near 3376 cm^−1^ represents the peak of intermolecular −OH stretching vibration. It is mainly attributed to the stretching vibration of the molecularly associated hydroxyl group in the polymer, indicating the existence of intermolecular hydrogen bonds in the sample. The absorption peak near 2928 cm^−1^ may represent the C−H antisymmetric stretching vibration peak on the saturated carbon atom. The strongest peak at 1655 cm^−1^ is the stretching vibration peak of protein amide Ⅰ with C=O, and the absorption peak at 1542 cm^−1^ is the N−H stretching vibration peak of amide Ⅱ. The absorption peak near 1408 cm^−1^ is the bending vibration absorption of C−H on saturated carbon, whereas the peak at 1300−600 cm^−1^ is the fingerprint region, because in this band, single-bond stretching vibration and the molecular structure due to bond deformation vibration generate a complex spectrum. A slight difference in the molecular structure results in a slight difference in the absorption peak. The strongest broad peak at 925−1300 cm^−1^ is generated by C−O stretching vibration. Aside from the differences in the relative intensity of the peaks representing various components, there was little difference found in their shapes and positions.

#### 3.4.2. X-ray Diffraction (XRD) Analysis

**Figure 5:**
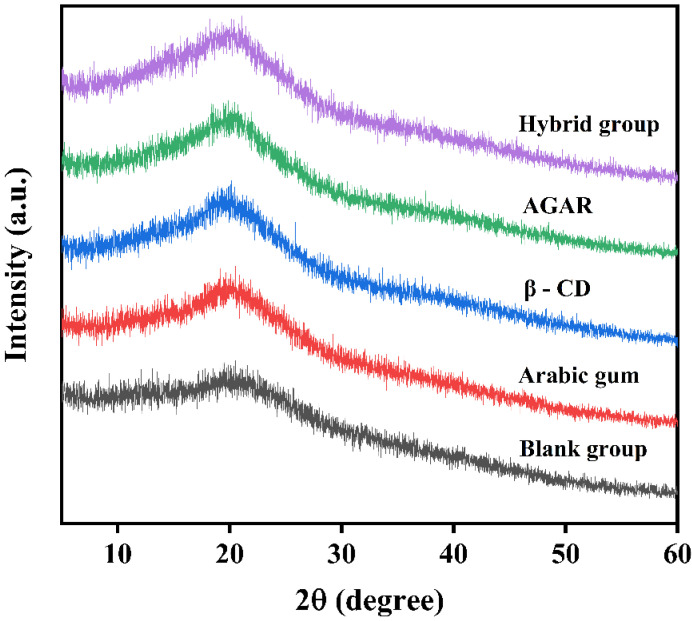
X-ray diffraction spectra of milk tea with different stabilizers.

XRD analysis is based on the X-ray diffraction of single crystals. A crystal is a three-dimensional structure formed by the regular periodic arrangement of atoms, molecules or ions. X-ray diffraction involves projection onto a crystal and scattering by atoms. The atoms are arranged periodically in the crystal, and the phase relationship between the scattered waves is fixed. The strength of the diffraction peak reflects the degree of crystallization of the sample under testing. The scattered X-rays are phase-enhanced in specific directions, resulting in characteristic diffraction phenomena corresponding to the crystalline structure [37]. The X-ray diffraction spectra of milk tea with different stabilizers are shown in Figure 5 below.

As shown in the figure, a wide diffraction peak of the composite system was detected at about 20°. The milk tea system was an amorphous structure. Compared with the blank group, the diffraction peak intensity of the milk tea with stabilizer was increased. The crystallinity of each sample was calculated according to the XRD pattern described in Section 2.3.2. We could observe the changes in the crystal structure of the milk tea treated with different stabilizers more clearly. The crystallinity of the milk tea system in the blank group was 23.49%, and the crystallinities after treatment with acacia gum, β-CD and agar were 26.40%, 30.49% and 28.76%, respectively. The crystallinity of the milk tea with response surface stabilizer was 33.16%, i.e., compared with the blank group, the increase was 9.67%. This shows that the orderly crystallization of milk tea is facilitated by the interaction between the three stabilizers at the right ratio, which results in an orderly structure [38].

#### 3.4.3. SEM Analysis of Microstructure

**Figure 6:**
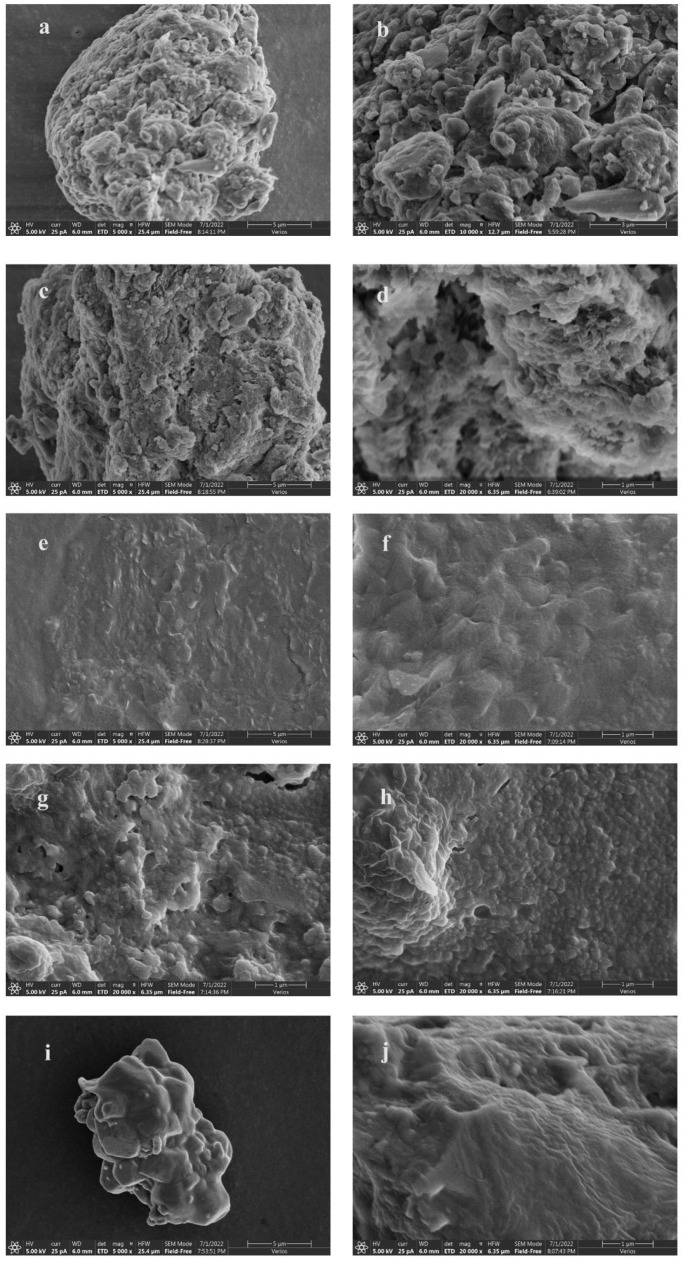
SEM microstructures of milk tea with different stabilizers. (**a**,**b**): Blank group; (**c**,**d**): Arabic gum group; (**e**,**f**): β-CD group; (**g**,**h**): Agar group; (**i**,**j**): Compound group.

A scanning electron microscope (SEM) is a kind of microscopic technology falling between a transmission electron microscope (TEM) and optical microscope. It uses secondary electron signal imaging to observe the surface morphology of samples. The polyphenol–protein complex in milk tea primarily contributes to its flocculation. Flocculation can be partially reduced by adding different concentrations and types of stabilizers into the milk tea system. Figure 6 shows the SEM microstructures of the milk tea supplemented with different stabilizers.

When no stabilizer (Figure 6a,b) was added to the milk tea, complex complexes were formed through the interaction between polyphenols in the tea and proteins in the milk, which increased the likelihood of flocculation, turbidity, precipitation and other phenomena. The addition of Arabic gum (Figure 6c,d) to milk tea resulted in a smoother and tightly connected surface, suggesting a good surface activity of acacia gum and the encapsulation of unstable ingredients on the surface of milk tea. Some studies have shown that β-CD (Figure 6e,f) interferes with the interaction between α-amylase and proanthocyanidins. β-CD may also act as a stabilizer bound to polyphenols via a competitive mechanism, thus reducing the interaction between proteins and polyphenols [39]. It can be intuitively observed from the figure that the milk tea supplemented with β-CD has a uniform texture and is relatively smooth. The structures coated on the surface of the milk tea are large oval molecules that are closely connected with each other. Agar (Figure 6g,h), as a thickening agent, contains many hydrophilic groups. After hydration with water molecules, these groups are highly dispersed in the water. A single-phase, uniformly dispersed system with a high viscosity, dense fish-scale structure and orderly arrangement was formed through the addition of 0.04% acacia gum, 0.02% β-CD and 0.03% agar to milk tea (Figure 6i,j). It is clear that the polyphenol–protein complex was coated, compared with the blank (Figure 6a,b). The surface was smooth and orderly, and the three stabilizers complemented each other.

## 4. Conclusions

The addition of 0.04% Arabic gum, 0.02% β-CD and 0.03% agar can regulate the flocculation of milk tea. These three stabilizers show synergistic effects when combined with the response surface. Through the characterization experiment, we found that milk tea has common absorption peaks in the presence of different stabilizers, without significant differences in the peak shape or location. Furthermore, the diffraction peak of the milk tea system is about 20°, suggesting an amorphous structure. The crystallinity is enhanced when single and compound stabilizers are used. The crystallinity of the milk tea in the compound group was 33.16%, which was higher than that of the blank group (9.67%). The ordered crystallization of milk tea was significantly enhanced by the addition of compound stabilizers, followed by β-CD. These results indicate that the structure of the milk tea system is complex, while its flocculation can be regulated by the addition of complex stabilizers. Single and compound stabilizers regulate the flocculation of milk tea and improve the order and stability of the structure. The effect of the complex stabilizer is better, possibly because the steric hindrance of the complex stabilizer in milk tea and the perfect cavity size reduce the possibility of polyphenol–protein binding. However, the detailed mechanism of milk tea flocculation remains to be studied; thus, further studies are needed to reveal these mechanisms.

## Data Availability

The datasets generated for this study are available on request to the corresponding author.

## Supplementary Materials

The following supporting information can be downloaded at: https://www.mdpi.com/article/10.3390/foods12071484/s1, Table S1: Factor level table for stability test of milk tea; Table S2: Box—Behnken design milk tea stabilizer combination formula and results; Table S3: Variance analysis of response surface test results.

## References

[1] Zhu M.Z., Wen B., Wu H., Li J., Lin H., Li Q., Li Y., Huang J., Liu Z. (2019). The Quality Control of Tea by Near-Infrared Reflectance (NIR) Spectroscopy and Chemometrics. J. Spectrosc..

[2] Sert D., Mercan E., Kilinc M. (2022). Powder Flow Behaviour, Functional Characteristics and Microstructure of Whole Milk Powder Produced from Cow and Buffalo Milk Mixtures. Int. Dairy J..

[3] Czubinski J., Dwiecki K. (2017). A Review of Methods Used for Investigation of Protein-Phenolic Compound Interactions. Int. J. Food Sci. Technol..

[4] Jongberg S., Andersen M.L., Lund M.N. (2020). Characterisation of Protein-Polyphenol Interactions in Beer during Forced Aging. J. Inst. Brew..

[5] Tosif M.M., Najda A., Bains A., Krishna T.C., Chawla P., Dyduch-Sieminska M., Klepacka J., Kaushik R. (2021). A Comprehensive Review on the Interaction of Milk Protein Concentrates with Plant-Based Polyphenolics. Int. J. Mol. Sci..

[6] Ozdal T., Capanoglu E., Altay F. (2013). A Review on Protein-Phenolic Interactions and Associated Changes. Food Res. Int..

[7] Sommer S., Weber F., Harbertson J.F. (2019). Polyphenol-Protein-Polysaccharide Interactions in the Presence of Carboxymethyl Cellulose (CMC) in Wine-Like Model Systems. J. Agric. Food Chem..

[8] Song Y., Yoo S.H. (2017). Quality Improvement of a Rice-Substituted Fried Noodle by Utilizing the Protein-Polyphenol Interaction between a Pea Protein Isolate and Green Tea (*Camellia sinensis*) Extract. Food Chem..

[9] Jauregi P., Guo Y.C., Adeloye J.B. (2021). Whey Proteins-Polyphenols Interactions Can Be Exploited to Reduce Astringency or Increase Solubility and Stability of Bioactives in Foods. Food Res. Int..

[10] Thongkaew C., Gibis M., Hinrichs J., Weiss J. (2014). Polyphenol Interactions with Whey Protein Isolate and Whey Protein Isolate-Pectin Coacervates. Food Hydrocoll..

[11] Xiao N.H., He W., Zhao Y., Yao Y., Xu M.S., Du H.Y., Wu N., Tu Y.G. (2021). Effect of PH and Xanthan Gum on Emulsifying Property of Ovalbumin Stabilized Oil-in Water Emulsions. LWT-Food Sci. Technol..

[12] Zheng Y.X., Sun W.X., Yang W.H., Chen S.G., Liu D.H., Tian J.H., Ye X.Q. (2020). The Influence of Xanthan Gum on Rheological Properties and In Vitro Digestibility of Kudzu (*Pueraria lobata*) Starch. Starch-Starke.

[13] Brenner T., Tuvikene R., Fang Y.P., Matsukawa S., Nishinari K. (2015). Rheology of Highly Elastic Iota-Carrageenan/Kappa-Carrageenan/Xanthan/Konjac Glucomannan Gels. Food Hydrocoll..

[14] Sadaquat H., Akhtar M. (2020). Comparative Effects of β-Cyclodextrin, HP-β-Cyclodextrin and SBE7-β-Cyclodextrin on the Solubility and Dissolution of Docetaxel via Inclusion Complexation. J. Incl. Phenom. Macrocycl. Chem..

[15] Fekete E., Bella E., Csiszar E., Moczo J. (2019). Improving Physical Properties and Retrogradation of Thermoplastic Starch by Incorporating Agar. Int. J. Biol. Macromol..

[16] Goncharuk V.V., Dubrovina L.V. (2020). Rheological Properties and Water-Retaining Power of Agar Hydrogels with Carboxymethyl Cellulose. Russ. J. Appl. Chem..

[17] Ishii T., Matsumiya K., Aoshima M., Matsumura Y. (2018). Microgelation Imparts Emulsifying Ability to Surface-Inactive Polysaccharides-Bottom-up vs Top-down Approaches. NPJ Sci. Food.

[18] Hussain M., Bakalis S., Gouseti O., Zahoor T., Anjum F.M., Shahid M. (2015). Dynamic and Shear Stress Rheological Properties of Guar Galactomannans and Its Hydrolyzed Derivatives. Int. J. Biol. Macromol..

[19] Gao F., Liu X.M., Chen W.X., Guo W., Chen L.M., Li D.M. (2018). Hydroxyl Radical Pretreatment for Low-Viscosity Sodium Alginate Production from Brown Seaweed. Algal Res..

[20] Seo S.Y., Lee G.H., Lee S.G., Jung S.Y., Lim J.O., Choi J.H. (2012). Alginate-Based Composite Sponge Containing Silver Nanoparticles Synthesized in Situ. Carbohydr. Polym..

[21] Watanabe T., Kawai T., Nonomura Y. (2018). Effects of Fatty Acid Addition to Oil-in-Water Emulsions Stabilized with Sucrose Fatty Acid Ester. J. Oleo Sci..

[22] Zhao X., Huang B.Y., El-Aooiti M., Rousseau D. (2018). Demulsification to Control Solute Release from Pickering Crystal Stabilized Water-in-Oil Emulsions. J. Colloid Interface Sci..

[23] Wijarnprecha K., de Vries A., Santiwattana P., Sonwai S., Rousseau D. (2019). Microstructure and Rheology of Oleogel-Stabilized Water-in-Oil Emulsions Containing Crystal-Stabilized Droplets as Active Fillers. LWT-Food Sci. Technol..

[24] Zhang W.H., Yang Y.Y., Lv T., Fan Z.Y., Xu Y.Q., Yin J.F., Liao B.W., Ying H., Ravichandran N., Du Q.Z. (2017). Sucrose Esters Improve the Colloidal Stability of Nanoethosomal Suspensions of (-)-Epigallocatechin Gallate for Enhancing the Effectiveness against UVB-Induced Skin Damage. J. Biomed. Mater. Res. Part B.

[25] Korir M.W., Wachira F.N., Wanyoko J.K., Ngure R.M., Khalid R. (2014). The Fortification of Tea with Sweeteners and Milk and Its Effect on in Vitro Antioxidant Potential of Tea Product and Glutathione Levels in an Animal Model. Food Chem..

[26] Ilyasoglu H., Arpa T.E. (2017). Effect of Brewing Conditions on Antioxidant Properties of Rosehip Tea Beverage: Study by Response Surface Methodology. J. Food Sci. Technol.-Mysore.

[27] Wagoner T.B., Ward L., Foegeding E.A. (2015). Using State Diagrams for Predicting Colloidal Stability of Whey Protein Beverages. J. Agric. Food Chem..

[28] Li Y.Q., Fan L.P. (2020). Comparative Studies on the Stabilization of Flos Sophorae Immaturus Beverages by Various Hydrocolloids. LWT-Food Sci. Technol..

[29] Ahmadi S.F., Nasirpour A., Goli S.A.H., Riahi E. (2018). Effect of Heat Treatment and Solution Preparation Procedure on Colloidal Stability of Whey Protein Sour Cherry Beverage. Int. J. Dairy Technol..

[30] Omranian S.R., Hamzah M.O., Yee T.S., Hasan M.R.M. (2020). Effects of Short-Term Ageing Scenarios on Asphalt Mixtures’ Fracture Properties Using Imaging Technique and Response Surface Method. Int. J. Pavement Eng..

[31] Dima S.O., Panaitescu D.M., Orban C., Ghiurea M., Doncea S.M., Fierascu R.C., Nistor C.L., Alexandrescu E., Nicolae C.A., Trica B. (2017). Bacterial Nanocellulose from Side-Streams of Kombucha Beverages Production: Preparation and Physical-Chemical Properties. Polymers.

[32] Medina-Jaramillo C., Bernal C., Fama L. (2020). Influence of Green Tea and Basil Extracts on Cassava Starch Based Films as Assessed by Thermal Degradation, Crystalline Structure, and Mechanical Properties. Starch-Starke.

[33] Mitic A.D., Gasic J.Z., Barac R.G., Radenkovic G.S., Sunaric S.M., Popovic J.Z., Nikolic M.M. (2020). Ultrastructural Changes in the Cemento-Enamel Junction Caused by Acidic Beverages: An in Vitro Study. Microsc. Res. Tech..

[34] Rocha C.M.R., Souza H.K.S., Magalhaes N.F., Andrade C.T., Goncalves M.P. (2014). Rheological and Structural Characterization of Agar/Whey Proteins Insoluble Complexes. Carbohydr. Polym..

[35] Hu B., Han L.Y., Kong H.L., Nishinari K., Phillips G.O., Yang J.X., Fang Y.P. (2019). Preparation and Emulsifying Properties of Trace Elements Fortified Gum Arabic. Food Hydrocoll..

[36] Zhang R.B., Liu T., Zhang Y.M., Cai Z.L., Yuan Y.Z. (2020). Preparation of Spent Fluid Catalytic Cracking Catalyst-Metakaolin Based Geopolymer and Its Process Optimization through Response Surface Method. Constr. Build. Mater..

[37] Liu K., Li Q.M., Zha X.Q., Pan L.H., Bao L.J., Zhang H.L., Luo J.P. (2019). Effects of Calcium or Sodium Ions on the Properties of Whey Protein Isolate-Lotus Root Amylopectin Composite Gel. Food Hydrocoll..

[38] Liu Y.R., Li B.F., Zhang K., Li J.W., Hou H. (2019). Novel Hard Capsule Prepared by Tilapia (*Oreochromis niloticus*) Scale Gelatin and Konjac Glucomannan: Characterization, and in Vitro Dissolution. Carbohydr. Polym..

[39] Soares S., Mateus N., de Freitas V. (2012). Carbohydrates Inhibit Salivary Proteins Precipitation by Condensed Tannins. J. Agric. Food Chem..

