# Supplementary material for: Reducing the Flocculation of Milk Tea Using Different Stabilizers to Regulate Tea Proteins
Source: Foods. 2023 Apr 1;12(7):1484. doi: 10.3390/foods12071484 (PMC10094277; doi:10.3390/foods12071484)
Supplement: Supplementary file 1 [file foods-12-01484-s001.zip › foods-2258625-supplementary.pdf]

Table S1. Factor level table for stability test of milk tea

Levels	Factors		
	X_1（%）	X_2（%）	X_3（%）
-1	0.02	0.02	0.02
0	0.03	0.03	0.03
1	0.04	0.04	0.04

Table S2. Box - Behnken design milk tea stabilizer combination formula and results

Run	Arabic gum (%)	β-CD (%)	AGAR (%)	Stability coefficient (R)
1	0.02	0.03	0.02	0.71577
2	0.03	0.03	0.03	0.75798
3	0.04	0.02	0.03	0.76393
4	0.03	0.04	0.04	0.70619
5	0.03	0.03	0.03	0.76338
6	0.02	0.03	0.04	0.68193
7	0.03	0.03	0.03	0.77188
8	0.04	0.03	0.02	0.72977
9	0.03	0.02	0.04	0.748
10	0.03	0.04	0.02	0.74308
11	0.04	0.03	0.04	0.7479
12	0.03	0.02	0.02	0.74384
13	0.03	0.03	0.03	0.76142
14	0.03	0.03	0.03	0.75689
15	0.02	0.04	0.03	0.70784
16	0.04	0.04	0.03	0.72093
17	0.02	0.02	0.03	0.71247

Table S3. Variance analysis of response surface test results

Source	Sum of Squares	Degree of freedom	Mean Square	F-value	p-value Prob > F	Comment
Model	0.010	9	1.153×10^{-3}	38.27	< 0.0001	significant
X_1	2.611×10^{-3}	1	2.611×10^{-3}	86.66	< 0.0001	
X_2	1.017×10^{-3}	1	1.017×10^{-3}	33.75	0.0007	
X_3	2.934×10^{-4}	1	2.934×10^{-4}	9.74	0.0168	
X_1X_2	3.680×10^{-4}	1	3.680×10^{-4}	12.21	0.0101	
X_1X_3	6.753×10^{-4}	1	6.753×10^{-4}	22.41	0.0021	
X_2X_3	4.214×10^{-4}	1	4.214×10^{-4}	13.98	0.0073	
X_1^2	2.896×10^{-3}	1	2.896×10^{-3}	96.13	< 0.0001	
X_2^2	4.038×10^{-4}	1	4.038×10^{-4}	13.40	0.0081	
X_3^2	1.251×10^{-3}	1	1.251×10^{-3}	41.54	0.0004	
Residual	2.109×10^{-4}	7	3.013×10^{-5}			
Lack of Fit	6.921×10^{-5}	3	2.307×10^{-5}	0.65	0.6226	not significant
Pure Error	1.417×10^{-4}	4	3.542×10^{-5}			
Cor Total	0.011	16				
R^2					0.9801	
R^2_{Adj}					0.9545	
